# Targeting sites of inflammation: intercellular adhesion molecule-1 as a target for novel inflammatory therapies

**DOI:** 10.3389/fphar.2013.00127

**Published:** 2013-10-04

**Authors:** Susan Hua

**Affiliations:** The School of Biomedical Sciences and Pharmacy, The University of Newcastle, CallaghanNew South Wales, Australia

**Keywords:** inflammation, targeted drug delivery, ICAM-1, cell adhesion molecules, liposomes

## Abstract

Targeted drug delivery to sites of inflammation will provide effective, precise, and safe therapeutic interventions for treatment of diverse disease conditions, by limiting toxic side effects and/or increasing drug action. Disease-site targeting is believed to play a major role in the enhanced efficacy observed for a variety of drugs when formulated inside lipid vesicles. This article will focus on the factors and mechanisms involved in drug targeting to sites of inflammation and the importance of cell adhesion molecules, in particular intercellular adhesion molecule-1, in this process.

## INTRODUCTION

Targeted delivery of therapeutics to sites of inflammation is an important goal. The endothelium represents a key target for pharmacological interventions in many disease conditions, including rheumatological, cardiovascular, hematological, pulmonary, and oncological (Koning et al., 2002; Metselaar and Storm, 2005; Muro and Muzykantov, 2005; Ding et al., 2006). The goal of endothelial targeting is to achieve specific and safe delivery of a drug to, into, or across endothelial cells, in order to localize effects in the lumen, desired intracellular endothelial cell compartments, or extravascular space, thereby improving pharmacological interventions. However, due to their lack of affinity to the endothelium, only a small fraction of injected therapeutics binds to endothelial cells (Ding et al., 2006). Progress in understanding disease mechanisms provides better selection of drugs for endothelial interventions and a deeper insight into designing drug delivery carriers to target inflammatory specific destinations.

## DRUG TARGETING TO SITES OF INFLAMMATION

In inflamed tissues, the permeability of the vasculature is often increased to the extent that particulate carriers, which are normally excluded from these tissues, can extravasate and localize in the tissue interstitial space. Endothelial cells also start to express several types of adhesion molecules: the selectins, the integrins, and the immunoglobulins, which mediate recruitment of leukocytes into the inflamed tissue (Koning et al., 2002; Metselaar and Storm, 2005; Ding et al., 2006). Furthermore, the process of angiogenesis (formation of new blood vessels from pre-existing vasculature) may occur in several chronic inflammatory disorders, such as rheumatoid arthritis, psoriasis, and inflammatory bowel disease (Koning et al., 2002; Metselaar and Storm, 2005). In this complex cascade of events, numerous cell-surface receptors, adhesion molecules, and growth factors are involved, which may serve as potential targets for therapeutic intervention.

Targeted intervention in inflammatory disease at the vascular endothelial cell (VEC) level has great potential. Rational design of such drug delivery systems includes: (1) selection of proper target determinants on endothelial surfaces, such as cell adhesion molecules (CAMs); (2) production of affinity ligands useful for targeting, such as affinity peptides, antibodies, or their fragments; (3) selection and adopting of suitable delivery vehicles, such as liposomes; and (4) formulation of drug delivery system with optimal targeting and therapeutic features (Ding et al., 2006). Specific drug delivery should concentrate the drug at the targeted site, increasing efficacy, and also decreasing side effects in other tissues (Willis and Forssen, 1998; Maruyama, 2002). This concept is particularly attractive in cancer therapy, where the dose a patient can tolerate is limited due to high toxicity to non-target cells. Targeted delivery to tumor tissue may allow the use of lower drug-concentrations. Moreover, targeting therapeutic agents to the vasculature of tumors offer additional advantages; in particular blood vessels are more readily accessible to intravenously administered therapy than tumor cells (Bendas, 2001). In the same respects, targeted drug delivery of opioid analgesics to peripheral opioid receptors upregulated at sites of inflammation will significantly alleviate nociception without the central opioid mediated side effects (Hua and Cabot, 2013).

### TARGETING ADHESION MOLECULES

Adhesion molecules are glycoproteins expressed on cell surfaces, where they mediate the contact between two cells (both homotypic and heterotypic interactions) or between cells and the extracellular matrix. They are essential for the regulation of immune cell responses and migration of inflammatory cells from the blood vessels into inflamed tissues (Bloemen et al., 1995; Mastrobattista et al., 1999). In fact, the expression of particular CAMs [e.g., intercellular adhesion molecule-1 (ICAM-1), E-selectin, P-selectin, vascular cell adhesion molecule-1 (VCAM-1)] are locally induced or enhanced at areas of inflammation (Bloemen et al., 1995; Spragg et al., 1997; Mastrobattista et al., 1999; Koning et al., 2002; Sakhalkar et al., 2003; Muro and Muzykantov, 2005; Voinea et al., 2005). Upregulated and/or overexpressed CAMs can be found in a multitude of clinical diseases where inflammation and immune cells are involved (e.g., ischemia-reperfusion injury, transplant rejection, and inflammatory diseases of the cardiovascular system, skin, kidneys, gastrointestinal tract, brain, and liver; Bloemen et al., 1995; Spragg et al., 1997; Mastrobattista et al., 1999; Koning et al., 2002; Muro and Muzykantov, 2005). Additionally, tumor cells use adhesion molecules to grow and spread throughout the body (Janssen et al., 2003).

Of particular note is the implication of CAMs in the pathogenesis of several rheumatic diseases. For example, rheumatoid arthritis is a chronic inflammatory disease in which adhesion molecules play an important role in the invasion of leukocytes into synovial tissues, leading to tissue damage (Mojcik and Shevach, 1997; Metselaar et al., 2003). Not only have increased expression of E-selectin, VCAM-1, and ICAM-1 been found on the vascular endothelium of synovial tissues, but immunohistochemical studies have shown elevated levels of adhesion molecule expression in ongoing inflammatory lesions (Mojcik and Shevach, 1997; Koning et al., 2002). Although reduction or blockade of the expression or function of a specific CAM is a possible therapeutic way to diminish infiltration and/or activation of inflammatory immune cells in order to reduce inflammation, this approach is complicated by the fact that most types of adhesion molecules are expressed on more than one cell type, that most cells express more than one adhesion molecule on their surface, and that several molecules can function as a ligand for a single adhesion molecule (Mojcik and Shevach, 1997; Koning et al., 2002). Importantly, blockade of CAMs can interfere with functions of immune cells essential for host defense (Mojcik and Shevach, 1997; Koning et al., 2002). Induction and/or increased expression of certain CAMs at inflammatory loci associated with various diseases offers opportunities for the development of new therapeutic strategies aimed toward selective drug-targeting. Adhesion molecules represent an easily accessible target molecule for therapeutics circulating in the blood compartment (Bloemen et al., 1995; Koning et al., 2002; Metselaar and Storm, 2005; Muro and Muzykantov, 2005; Ding et al., 2006).

### INTERCELLULAR ADHESION MOLECULE-1

Intercellular adhesion molecules (ICAMs) are structurally related transmembrane glycoproteins of the immunoglobulin supergene family and are ligands for the β2 integrin molecules present on leukocytes (Almenar-Queralt et al., 1995; Hubbard and Rothlein, 2000). Of the five ICAMs identified, ICAM-1 is the most extensively studied (Koning et al., 2002; Muro and Muzykantov, 2005). ICAM-1 specifically participates in trafficking of inflammatory cells, in leukocyte effector functions, in adhesion of antigen-presenting cells to T lymphocytes, in microbial pathogenesis, and in signal transduction pathways through outside-in signaling events (Almenar-Queralt et al., 1995; Hubbard and Rothlein, 2000; Muro and Muzykantov, 2005). This adhesion molecule is localized to both the apical and basolateral surface of endothelial cells, making it ideally positioned to facilitate transendothelial migration of leukocytes (Almenar-Queralt et al., 1995). In fact, ICAM-1 (along with VCAM-1) is considered to represent the most important adhesion molecule for leukocyte recruitment to inflamed sites (Koning et al., 2002). Additionally, ICAM-1 has been shown to exist in a soluble form in circulation, which results from proteolytic cleavage mediated by neutrophil proteases (leukocyte elastase and cathepsin G) in a process independent of ICAM-1 surface density (Muro and Muzykantov, 2005).

Intercellular adhesion molecule-1 is widely distributed and expressed constitutively at low levels on leukocytes, VECs, fibroblasts, and epithelial cells. Although ICAM-1 is present in several cell types, the level of expression is orders of magnitude lower than that of VECs (Almenar-Queralt et al., 1995; Scholz et al., 1996; Mojcik and Shevach, 1997; Hubbard and Rothlein, 2000; Koning et al., 2002; Muro et al., 2003b, 2005; Muro and Muzykantov, 2005). Stimulation of a variety of cells with inflammatory cytokines such as interleukin-1 (IL-1), tumor necrosis factor-α (TNF-α), and interferon-γ (IFN-γ) has been documented to increase ICAM-1 expression on multiple cell types (Almenar-Queralt et al., 1995; Scholz et al., 1996; Hubbard and Rothlein, 2000; Muro et al., 2003b, 2005; Muro and Muzykantov, 2005). Strong upregulation of ICAM-1 is observed under inflammatory conditions within 24 h (Scholz et al., 1996). In contrast to the selectins, which are rapidly down regulated after induction, ICAM-1 and VCAM-1, once upregulated, remain on the cell surface for more than 48 h (Koning et al., 2002). Information on the internalization capacity of ICAM-1 is, however, rather contradictory (Koning et al., 2002). Some authors report the total absence of internalization on TNF-activated human umbilical vein endothelial cells (HUVEC) or a rather slow process of internalization by HUVECs, whereas others report on (rapid) internalization of ICAM-1-binding peptides by lymphocytes, antibody-targeted liposomes or poly lactic-co-glycolic acid (PLGA) nanoparticles by epithelial cells (Koning et al., 2002; Chittasupho et al., 2009). In fact, it has been reported that ICAM-1 internalization levels are practically indistinguishable from background (<10% of surface expressed ICAM-1; Muro and Muzykantov, 2005). These contradictory findings may not only be attributed to the difference in cell types and targeting ligands used, but also to the timeframe of the study (Koning et al., 2002). It is therefore of great importance to test the internalization capacity of the developed drug delivery system. Targeting to non- or slow-internalizing epitopes may be of specific interest for drugs that work at the luminal site of the VECs, whereas fast-internalizing epitopes are interesting for drugs with an intracellular address (Koning et al., 2002; Muro and Muzykantov, 2005).

Several adhesion molecules involved in the leukocyte adhesion cascade, in principle, comply with the requirements for achieving targeted delivery of drugs into VECs. However of these, ICAM-1 represents an attractive target since it is a high-density determinant stably exposed from the endothelial surface, which is upregulated and functionally involved in inflammation (Almenar-Queralt et al., 1995; Scholz et al., 1996; Mojcik and Shevach, 1997; Hubbard and Rothlein, 2000; Koning et al., 2002; Muro et al., 2003b, 2005; Muro and Muzykantov, 2005). In particular, ICAM-1 seems to be well suited for drug targeting to the luminal surface, due to ineffective internalization of either monomolecular or large anti-ICAM conjugates (Muro and Muzykantov, 2005). Potentially this will allow extravasation of the delivery carrier across the endothelial cells and release of the therapeutic drug specifically into the inflammatory site of action (Metselaar and Storm, 2005; Ding et al., 2006). This feature distinguishes ICAM from other similarly prevalent endothelial determinants all of which are rapidly internalized, therefore leading to early release of the drug within the endothelial cells themselves (Koning et al., 2002; Muro and Muzykantov, 2005).

Antibodies to CAMs are being explored as therapeutics and delivery carriers in cell cultures, animal models, and early clinical studies (Muro et al., 2005). A small number of studies have demonstrated the validity of such an approach, in particular showing specific binding and drug delivery to VECs *in vitro* and *in vivo* (Bloemen et al., 1995; Spragg et al., 1997; Bendas et al., 1998; Mastrobattista et al., 1999; Kessner et al., 2001; Jaafari and Foldvari, 2002b; Koning et al., 2002; Asgeirsdottir et al., 2003; Everts et al., 2003; Murciano et al., 2003; Muro et al., 2003a, 2005; Muro and Muzykantov, 2005; Voinea et al., 2005; Ding et al., 2006). Presumably, the specific and strong upregulation of these CAMs at sites of inflammation still allows specific targeting to be observed. Therefore, ICAM-1 targeting seems attractive, as this CAM shows basal levels of expression on VECs in general, but is strongly upregulated on VECs at inflamed sites (Almenar-Queralt et al., 1995; Scholz et al., 1996; Mojcik and Shevach, 1997; Hubbard and Rothlein, 2000; Koning et al., 2002; Muro et al., 2003b, 2005; Muro and Muzykantov, 2005). These developments in drug targeting to VECs will result in increasing knowledge on the role of the endothelium in inflammatory disorders and will further improve clinical therapy.

### SELECTIVE INTERACTION WITH ICAM-1 AND UPTAKE BY TARGET CELLS

There are a number of potential modes of delivery of encapsulated therapeutics from ICAM-1 targeted carriers, which will affect its therapeutic availability and action. Contradicting results have been reported of the extent of internalization of ICAM-1-directed carriers by endothelial cells (Koning et al., 2002). The capacity of endothelial cells to uptake anti-CAM multimeric conjugates may depend on the size of the particles, with conjugates having diameters from 100 to 300 nm readily entering endothelial cells, whereas conjugates of larger size (500 nm to 1 μm) remained attached to the cell surface at 37°C (Murciano et al., 2003; Muro et al., 2003a; Muro and Muzykantov, 2005). The notion that small multimeric ligands can undergo internalization within endothelial cells by CAM-mediated endocytosis is of pharmacological and physiological relevance (Murciano et al., 2003; Muro et al., 2003a; Muro and Muzykantov, 2005). The signaling and cytoskeletal events involved in endothelial internalization of anti-CAM conjugates are similar to those triggered by CAM-clustering in course of leukocyte adhesion and transmigration (Muro and Muzykantov, 2005). This parallelism supports the notion that intracellular drug delivery mediated by anti-CAM conjugates may be further enhanced in inflammation and pathological conditions that activate such transduction pathways in endothelial cells (Muro and Muzykantov, 2005).

In addition to delivering therapeutic cargoes intracellularly or to the luminal surface to have an anti-inflammatory effect on the endothelial cells involved in inflammation (Przewlocki and Przewlocka, 2001; Stein et al., 2001), it is plausible for liposomes under pathological conditions to extravasate through the endothelial barrier directed by ICAM-1 on the surface of endothelial cells at sites of inflammation to release drugs within the extravascular tissue space (Oku and Namba, 1994; Vingerhoeds et al., 1994; Willis and Forssen, 1998; Koning et al., 2002; Antohe et al., 2004; Metselaar and Storm, 2005).

### FACTORS INFLUENCING TARGET ACCUMULATION IN INFLAMMATION

Drug targeting using liposomes as carriers holds much promise, especially in reducing toxicity and targeting delivery to pathological sites of inflammation (e.g., musculoskeletal conditions, infection, burns, tumors) that are characterized by increased vascular permeability (Oku and Namba, 1994; Vingerhoeds et al., 1994; Yuan et al., 1994; Thurston et al., 1998; Willis and Forssen, 1998; Klimuk et al., 1999; Laverman et al., 1999; Bendas, 2001; Koning et al., 2002; Maruyama, 2002; Antohe et al., 2004; Metselaar and Storm, 2005). Long-circulating liposomes are currently used in targeted drug delivery to tumors and inflammatory regions, and have shown impressive improvement of the therapeutic index of encapsulated drugs (Oku and Namba, 1994; Torchilin, 1994, 1996; Laverman et al., 1999; Bendas, 2001; Koning et al., 2002; Metselaar and Storm, 2005; Ding et al., 2006). For example, rats and mice with arthritis treated with a single intravenous (IV) injection of sterically stabilized liposomes (SL) containing prednisolone phosphate resulted in complete remission of paw inflammation for 1 week in comparison to free drug (Metselaar and Storm, 2005). Mechanistic studies showed that the increased therapeutic benefit was a result of selective joint targeting (Metselaar and Storm, 2005).

Within inflamed tissues the permeability of the vasculature is often increased to the extent that particulate carriers, which are normally excluded from these tissues, can extravasate and localize in the tissue interstitial space (Antohe et al., 2004; Metselaar and Storm, 2005). This selective accumulation and increase in drug concentration at inflamed target sites is due to the so-called enhanced permeability and retention (EPR) effect (Maruyama, 2002; Metselaar and Storm, 2005). Inflammation results in a dramatic change in blood vessel permeability as the capillary vasculature undergoes structural remodeling to allow leukocyte diapedesis into the peripheral tissue (Klimuk et al., 1999). The width of the tight junctional regions between endothelial cells *in vivo* has been reported to be from 12 to 20 nm (Antohe et al., 2004), however exposure of endothelial cells to inflammatory mediators increases permeability of the microvasculature, with the formation of gaps of up to 1 μm (Antohe et al., 2004). In fact, pore sizes ranging from 0.2 to 1.2 μm have been observed, though the size and number of pores are dependent upon the microenvironment of the pathological site (Klimuk et al., 1999; Antohe et al., 2004). Observations using fluorescence and electron microscopy have shown that SL can indeed extravasate beyond the endothelial barrier, mainly in postcapillary venules, with SL ranging from 100 to 200 nm in diameter having a higher probability of encountering the leaky vessels of the inflamed tissue (Willis and Forssen, 1998; Antohe et al., 2004; Metselaar and Storm, 2005). Leukocytes are able to open intercellular junctions of the endothelium monolayer by stimulating contraction of the endothelial cells or by causing a gap by passing between the cells (Antohe et al., 2004). It is therefore plausible that liposomal carriers may cross the monolayer in association with leukocytes or migrate independently across gaps formed in the monolayer by leukocyte migration (Klimuk et al., 1999; Sipkins et al., 2000; Antohe et al., 2004; Metselaar and Storm, 2005).

Currently, systemic liposome targeting strategies investigated are able to deliver no more than a few percent of the administered dose to their desired sites *in vivo* (Willis and Forssen, 1998). Although these formulations represent significant improvements over corresponding conventional drug therapies, much of the administered dose is still delivered to non-targeted tissues (Willis and Forssen, 1998). For example, a biodisposition study of polyethylene glycol (PEG)-coated lipid microspheres of indomethacin in arthritic rats reported an overall drug targeting efficiency of 7.5-fold higher than the conventional lipid microspheres (Palakurthi et al., 2005). The enhanced accumulation of the drug in the inflammatory tissue may be attributed to extravasation through the leaky vasculature and their possible uptake by circulating monocytes, which would subsequently be concentrated in the rheumatic joints (Palakurthi et al., 2005). Importantly, formulation as lipid microspheres drastically reduced the concentration of the drug in the brain (*C*_max_) from 1.73 to 0.69 μg/g of the tissue, thereby reducing central nervous system (CNS) adverse effects (Palakurthi et al., 2005). PEG-coated lipid microspheres further reduced the concentration to 0.58 μg/g (Palakurthi et al., 2005). The lower accumulation in sensitive non-target tissues (e.g., brain, kidneys) may be due to the reduced availability of the free drug in the blood (Palakurthi et al., 2005). It should be noted that the blood–brain barrier is often the rate-limiting factor in determining permeation of therapeutic drugs into the brain due to both physical (tight junctions) and metabolic (enzymes) barriers (Rousseau et al., 1999; Schmidt et al., 2003). Thus liposomal carriers are only able to localize in the brain more efficiently when this barrier has been altered (Rousseau et al., 1999; Schmidt et al., 2003; Palakurthi et al., 2005).

Attachment of target-specific ligands to the liposome surface (active targeting) has been shown to further enhance targeting to specific cells or tissues (Senior, 1987; Torchilin, 1994, 1996; Vingerhoeds et al., 1994; Willis and Forssen, 1998; Bendas, 2001; Maruyama, 2002; Ulrich, 2002). Targeting endothelial cells by exploiting cell-specific surface markers has been widely investigated *in vitro* (Bloemen et al., 1995; Willis and Forssen, 1998; Koning et al., 2002; Muro and Muzykantov, 2005; Ding et al., 2006). Liposomes have been modified with ligands that can selectively interact with E-selectin (Bendas et al., 1998; Kessner et al., 2001; Everts et al., 2003), ICAM-1 (Bloemen et al., 1995; Willis and Forssen, 1998; Mastrobattista et al., 1999; Sipkins et al., 2000; Jaafari and Foldvari, 2002a,b; Muro and Muzykantov, 2005; Ding et al., 2006) and VCAM-1 (Voinea et al., 2005) molecules that are upregulated on the surface of endothelial cells following activation by inflammatory signals. For example, P_0_-peptide-1 linked to liposome surfaces is capable of mediating the specific binding of liposomes to melanoma cells expressing high levels of ICAM-1, thus making it possible to target cancerous melanocytes in lymph nodes and skin melanomas (Jaafari and Foldvari, 2002b). Most of the work with liposomes in inflammatory disorders are based on imaging agents, with only few *in vivo* studies having been conducted using ligand-targeted liposomes incorporating therapeutic agents (Metselaar and Storm, 2005). For example, the biodistribution and target localization of E-selectin-targeted dexamethasone-containing liposomes was examined in a murine delayed-type hypersensitivity model, which reported enhanced uptake by activated endothelium at inflamed sites as compared with control tissue (Everts et al., 2003). Similarly, selective interaction with target cells following extravasation of targeted liposomes into the inflamed tissue has hardly been addressed *in vivo* (Metselaar and Storm, 2005). Boot et al. (2005) reported that the surface receptor CD134, specifically expressed by auto-aggressive T cells at sites of inflammation, could efficiently be targeted by liposomes modified with anti-CD134 antibody. It was observed that encapsulation of 5′-fluorodeoxyuridine dipalmitate in these liposomes could lead to inactivation of auto-aggressive T cells and amelioration of experimental arthritis (Boot et al., 2005). In addition, loperamide-encapsulated ICAM-1 targeted immunoliposomes have been shown to induce significant peripheral antinociceptive and anti-inflammatory activity in rats with complete Freund’s adjuvant-induced inflammation of the paw via an opioid receptor dependent mechanism (Hua and Cabot, 2013).

This phenomenon of disease-site targeting is believed to play a major role in the enhanced efficacy observed for a variety of drugs when formulated inside lipid vesicles (Oku and Namba, 1994; Vingerhoeds et al., 1994; Torchilin, 1996; Willis and Forssen, 1998; Bendas, 2001; Maruyama, 2002; Ulrich, 2002). Formulation of ICAM-1-directed sterically stabilized immunoliposomes (SIL) will not only allow prolonged circulation but also active targeting to sites of inflammation (Bloemen et al., 1995; Willis and Forssen, 1998; Koning et al., 2002; Muro and Muzykantov, 2005; Ding et al., 2006). Such drug carriers may escape from the gaps between adjacent endothelial cells and openings at the vessel termini during inflammation by passive convective transport and/or ligand-directed targeting (Antohe et al., 2004; Metselaar and Storm, 2005). It is also plausible that some liposomes can attach onto activated leukocytes undergoing diapedesis into inflammatory sites (Sipkins et al., 2000), as CAMs such as ICAM-1 are expressed not only on the surface of vascular endothelium and neurones, but also by activated T lymphocytes (Sipkins et al., 2000; Koning et al., 2002; Hua et al., 2006). This field of research of targeting therapeutics to sites of inflammation specific to pathological disease states will improve the efficacy of therapeutic agents and reduce the toxicity to other parts of the body.

## Conflict of Interest Statement

The author declares that the research was conducted in the absence of any commercial or financial relationships that could be construed as a potential conflict of interest.
